# Scalable Fleet Monitoring and Visualization for Smart Machine Maintenance and Industrial IoT Applications

**DOI:** 10.3390/s20154308

**Published:** 2020-08-02

**Authors:** Pieter Moens, Vincent Bracke, Colin Soete, Sander Vanden Hautte, Diego Nieves Avendano, Ted Ooijevaar, Steven Devos, Bruno Volckaert, Sofie Van Hoecke

**Affiliations:** 1 IDLab, Ghent University—imec, Technologiepark-Zwijnaarde 122, 9052 Gent, Belgium; Vincent.Bracke@UGent.be (V.B.); Colin.Soete@UGent.be (C.S.); Sander.VandenHautte@UGent.be (S.V.H.); Diego.NievesAvendano@UGent.be (D.N.A.); Bruno.Volckaert@UGent.be (B.V.); Sofie.VanHoecke@UGent.be (S.V.H.); 2 Corelab DecisionS, Flanders Make, Celestijnenlaan 300, 3001 Leuven, Belgium; Ted.Ooijevaar@FlandersMake.be (T.O.); Steven.Devos@FlandersMake.be (S.D.)

**Keywords:** fleet monitoring, bearing degradation, Cyber-Physical System, predictive maintenance, Industrial Internet of Things, Industry 4.0, accelerated lifetime testing

## Abstract

The wide adoption of smart machine maintenance in manufacturing is blocked by open challenges in the Industrial Internet of Things (IIoT) with regard to robustness, scalability and security. Solving these challenges is of uttermost importance to mission-critical industrial operations. Furthermore, effective application of predictive maintenance requires well-trained machine learning algorithms which on their turn require high volumes of reliable data. This paper addresses both challenges and presents the Smart Maintenance Living Lab, an open test and research platform that consists of a fleet of drivetrain systems for accelerated lifetime tests of rolling-element bearings, a scalable IoT middleware cloud platform for reliable data ingestion and persistence, and a dynamic dashboard application for fleet monitoring and visualization. Each individual component within the presented system is discussed and validated, demonstrating the feasibility of IIoT applications for smart machine maintenance. The resulting platform provides benchmark data for the improvement of machine learning algorithms, gives insights into the design, implementation and validation of a complete architecture for IIoT applications with specific requirements concerning robustness, scalability and security and therefore reduces the reticence in the industry to widely adopt these technologies.

## 1. Introduction

The Internet of Things (IoT) is the interconnection of computing devices embedded in assets or Things, enabling them to send and receive data [1]. Over the past decade, this paradigm has been applied to research areas such as smart environments [2], healthcare [3] and logistics [4]. In addition to these applications, IoT delivers a range of benefits to industry by enabling more efficient, optimized monitoring and controlling in a cost efficient manner. This application of IoT to industry is often described as Industry 4.0 or the Industrial Internet of Things (IIoT) [5]. The physical assets within an industrial environment are equipped with smart sensors, connecting them to the Internet and creating a Cyber-Physical System (CPS) of interconnected machines. These sensors collect and transmit valuable data about Key Performance Indicators (KPIs) which can be used by analytic and cognitive technologies to improve the overall performance of manufacturing plants by increasing the production or reducing its cost. In order to prevent the disruption of mission-critical industrial operations, the rise of IIoT has opened new challenges with regard to robustness, scalability and security. These requirements apply to each component within the overall IIoT architecture [6].

In the current trend toward digitalization and IIoT, manufacturing companies also increasingly rely on a range of technology platforms to help streamline their production and increase their productivity, among others by avoiding machine downtimes and the accompanying loss of turnover. Monitoring the condition of, for instance, motors, pumps, fans, turbines, gearboxes, and more specifically bearings and gears inside them, plays a vital role in the maintenance program of rotating machines. The most common KPIs for this category of machines are vibration and thermal signals [7]. Monitoring these KPIs leads to early fault detection, which is crucial for moving from a time-based Preventive Maintenance (PM) program to a condition-based Predictive Maintenance (PdM) strategy. This PdM strategy reduces unexpected machine downtime and unnecessary replacement of healthy machine parts, and the associated costs. In summary, through application of the IIoT paradigm and smart sensors, the condition of these machines can be constantly monitored and evaluated.

The resulting monitoring data can be used by state-of-the-art machine learning algorithms to calculate an estimate of the Remaining Useful Life (RUL) of the assets [8]. Targeted maintenance can then be scheduled based on these estimates. However, condition-based monitoring models require the availability of large and reliable data sets, for both training and validation purposes, that capture all variations observed in all potential operational conditions, the potential failure modes, the fault evolution and hence the lifetime of bearings. A supervised learning approach, for example, needs all this data to train the model and minimize incorrect classifications. Reliable benchmark datasets that capture the evolution of behavior of failing machines during its lifetime, under various operating conditions, are scarce and, for most companies, the investment cost of collecting this data, because it requires significant effort and a long time, does not outweigh the benefits they can reap. The resulting lack of data is a major hurdle for the adoption of condition-based monitoring in industry. Although recent advancements in sensor, acquisition and processing hardware have demonstrated cost-effective solutions [9,10], it is easier to quantify the cost than the economical benefit of this investment.

Flanders Make and imec, therefore, jointly developed a living lab, named the Smart Maintenance Living Lab. It is an open test and research platform consisting of seven identical drivetrain systems designed to monitor rolling-element bearings and accelerate the process of collecting data about their lifetime under diverse and variable operating conditions. The resulting well-documented data set can be used for the development, testing and validation of bearing fault diagnostic and prognostic methods. Moreover, it can be used in benchmark studies to compare the different methods.

The goal of this paper is to provide insights into the architecture of this living lab, and how this setup can enable condition-based machine maintenance. The paper starts with a high-level description of the living lab in Section 2. Section 3 contains an overview of the related research. The next three sections dive into the architectural details and essential building blocks, i.e., Section 4 explains the drivetrain subsystems, Section 5 goes into the Obelisk cloud platform, and Section 6 describes the dynamic dashboard. The paper is finalized with the results and a set of conclusions on the potential broader applicability of the living lab for machine condition monitoring in Section 7 and Section 8.

## 2. Smart Maintenance Living Lab

**Figure 1:**
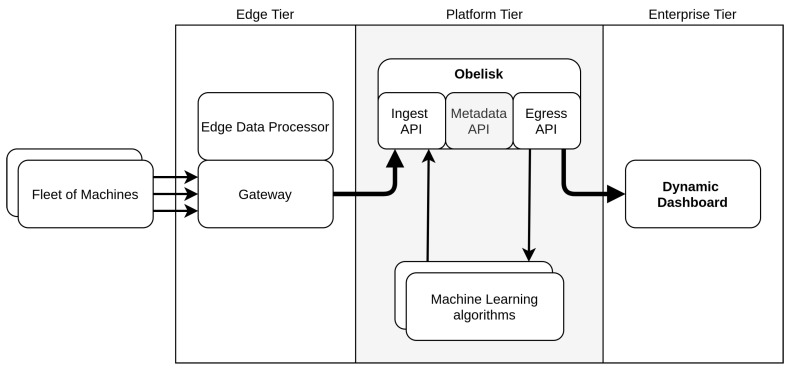
Presented scalable Industrial Internet of Things architecture.

The Smart Maintenance Living Lab is an open test and research platform for smart machine maintenance, from drivetrain subsystem, over the cloud, up to the dynamic dashboard. A schematic overview of its architecture is presented in Figure 1. Within the lab, seven identical drivetrain subsystems are installed, resembling a fleet. These machines are designed to perform accelerated testing of rolling-element bearings until their end-of-life. Moreover, they are equipped with different sensors to monitor the bearings throughout their lifetime and collect the resulting data. The platform aims to support the adoption of smart machine maintenance in industry through (i) the creation of a large, varied and well-documented dataset which can help with the development of accurate diagnostic and prognostic algorithms, and (ii) providing insights into the components required for and the technology choices made to come to an integrated end-to-end smart maintenance architecture, from monitored hardware to scalable cloud back-end to dynamic front-end.

For the architecture, an Industrial Internet Reference Architecture (IIRA), presented by the Industrial Internet Consortium (IIC) [11], was chosen. This three-tier architecture consists of an edge, platform and enterprise layer respectively.

The first tier contains the edge nodes, i.e., the sensors, actuators, and control systems connected to the physical machines. A cluster of interconnected nodes is called a proximity network. Each of these networks generally has an edge gateway to connect the nodes with the access network. The fleet of machines located in the Smart Maintenance Living Lab forms one proximity network consisting of seven edge nodes, i.e., the drivetrain systems. Each one of these subsystems is connected to the edge gateway. In addition to connectivity between the nodes and the access network, the gateway is also used to process the measured data at the edge. Diagnostic algorithms are used to reduce the high dimensionality of captured data by extracting a set of features. These diagnostic features are then transferred to the next tier, i.e., the platform tier.

The second layer, or platform tier, of an IIRA architecture has the goal of further processing and analyzing data from the edge tier, and sending control commands from the enterprise tier to the edge tier. In our setup, the most important component of this tier is Obelisk, our in-house cloud platform designed specifically for IoT applications that have scalable data needs. It handles the data ingest from the edge tier, ensures data persistence, supports data querying in various formats and enables stream processing. Furthermore, the platform tier includes analytic and machine learning services that process the collected data. The machine learning service in our platform tier estimates the RUL of the bearings for each drivetrain setup within the fleet. This estimation of RUL is based on the accelerometer and temperature data obtained from conditioning monitoring of the assets in the edge tier. Prognostic algorithms such as RUL prediction are key in enabling PdM.

The goal of the third and last tier, i.e., the enterprise tier, is to bundle domain-specific applications as well as decision support systems and to provide interfaces to end-users including operation specialists. In our enterprise tier, we provide a dynamic dashboard platform that allows end-users to build and adapt dashboards that communicate the sensor data captured in the fleet of machines, as well as the output of the RUL service, in an intuitive manner. This dashboard supports the operator in determining the appropriate follow-up actions, without hard-coded visualization configuration through automated sensor discovery and semantic reasoning to match data and results with the most appropriate visualisations.

Using this three-tier architecture, the living lab platform allows to collect large amounts of data about degrading bearings and the occurring failures during their lifetime under diverse and variable operating conditions. The resulting large data set can be used to:

- Test and benchmark the performance of existing software, e.g., diagnostic and prognostic algorithms, and hardware solutions such as intelligent sensors.
- Improve models and therefore develop, test and validate better diagnostics and prognostics algorithms.
- Demonstrate the potentials of condition-based fleet monitoring to improve machine diagnostics and prognostics by employing fleet data stored in the cloud environment.

Besides collecting a dataset with bearing faults under realistic and varied operating conditions, the living lab and this paper deals with two more research questions:

1. Design of a robust, scalable and secure platform for sensor data ingestion and stream processing;
2. Design of a dynamic, intuitive dashboard application that reduces the amount of manual configuration required to construct the desired sensor data visualizations.

All of these technologies offer great opportunities for the industry to optimize machine maintenance strategies. In this way, the living lab aims to bring wider adoption of condition-based monitoring technologies in industry and to support the development of new diagnostic and prognostic algorithms.

## 3. Related Work

As the Smart Maintenance Living Lab demonstrates a complete architecture for IIoT applications and, to the best of the authors’ knowledge is the only one doing so, related work is discussed at the different levels of the architecture: (i) the data collection at the edge tier, (ii) the data ingestion, persistence and stream processing platform as well as the analytic and cognitive machine learning algorithms in the platform tier and (iii) the dynamic dashboard for visualization and feedback.

The drive for adoption of smart maintenance in industry is dependent on intelligent condition monitoring models. These models require large, reliable data sets. Today, only few public data sets are available and they are recorded under limited operating conditions. The IEEE PHM 2012 Prognostic Challenge data set [12], for example, consists of 17 bearing runs under three different conditions of varying load and rotation speed. Each of these accelerated lifetime bearing runs is recorded on the same test bed setup. It was already apparent from the challenge that different operating conditions resulted in varying machine learning models. However, early results from the collected data also indicate a variability between different drivetrain subsystems [13]. Therefore, the data collected by the Smart Maintenance Living Lab on a fleet of seven machines can result in a more reliable data set that enables the generalization of algorithms created for smart maintenance use cases.

For the platform tier, various IoT middleware platforms, both commercial, e.g., Amazon Web Services IoT platform [14], Microsoft Azure IoT Hub [15], Google IoT Platform [16], and open-source alternatives, e.g., ThingSpeak [17] are available today. These platforms are well developed and maintained by large publicly traded companies or large online communities. They support numerous features with regard to security, data storage and communication protocols [18]. However, the use of these platforms creates a tightly coupled architecture to the chosen vendor, introducing constraints to the system and negatively impacting its modularity [19]. FIWARE [20] is technology and vendor agnostic as it relies on proven and widely adopted open-source standards and packages, alleviating its community of users from any lock-in constraint. However, as described by the authors in [20], they experienced scalability limitations due to the non cloud-based setup used. Obelisk [21], the IoT middleware platform presented in this paper for the platform tier, addresses these scalability issues.

Dashboard applications in the enterprise tier enable the remote and intuitive monitoring of the vast amount of data produced by the bearing lifetime test setups. Dynamic dashboard applications allow stakeholders, e.g., managers, system experts, customers, to build their own dashboards that visualize KPIs about the systems of their choice by selecting sensors and visualizations at will. Many off-the-shelf dynamic dashboards, such as Microsoft PowerBI [22], Tableau [23] and Google Data Studio [24], are either well integrated with classic data warehouses but do not integrate well with the vast amount of streaming sensor data and the streaming platform technology they require; or they require a lot of programming effort or manual configuration when combining the output of multiple sensors, e.g., Node-RED [25]. This manual configuration is required, on the one hand, at design time to instruct how to fetch data and bind it correctly to the available data processing and visualization components, and at runtime, on the other hand, there is still the burden for the user to select from a plethora of available sensors, data processing components and visualizations. In previous research [26], we proposed to wrap sensors, data processing components and visualizations as Web Things, i.e., APIs, that can be automatically discovered, called and combined, and this way to reduce the sensor and visualization choice overload by reasoning over the available metadata that the Web Things are annotated with. Within the Living Lab Smart Maintenance, this dynamic dashboard using semantic reasoning is integrated in the enterprise tier, on top of Obelisk.

## 4. Drivetrain Systems

As mentioned in Section 2, the edge tier consists of seven identical drivetrain systems [13]. A single setup comprises of a shaft with the test bearing, lubricated by an internal oil bath, and one support bearing on each side. The system is driven by a motor at a rotation of up to 3000 rpm and a radial load of up to a maximum of 10 kN is applied to the test bearing using a hydraulic cylinder. During the accelerated lifetime testing of the bearings, the rotational speed and radial load of each setup can be controlled, allowing it to operate under a variety of different conditions.

**Figure 2:**
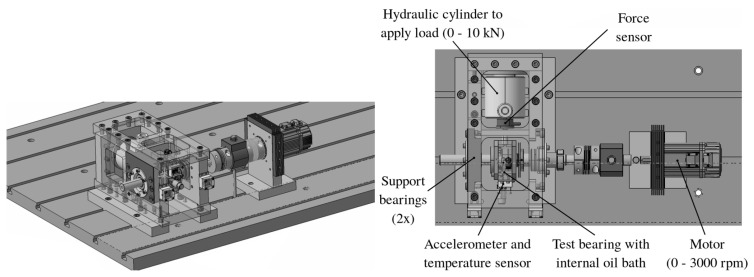
One of the seven setups that is used to accelerate the lifetime of bearings and allows to acquire data during the accumulation of operational bearing faults until end-of-life. Reprinted from IFAC-PapersOnLine, 52, Ooijevaar, T., et al., “Smart Machine Maintenance Enabled by a Condition Monitoring Living Lab”, 376–381, 2019, with permission from Elsevier.

As shown in Figure 2, each setup is equipped with an accelerometer, temperature sensor, load sensor and speed sensor for monitoring purposes. The speed and load applied to the system is controlled, and the measurements from these sensors are obtained, using an industrial Beckhoff control platform. The acquired sensor measurements are locally processed to reduce the high volume of data. An overview of the resulting measurements and diagnostic features is shown in Table 1.

These diagnostic features are transferred to the next tier, i.e., the platform tier, of which Obelisk is the most important component.

## 5. Obelisk: Scalable Cloud Platform for IoT Applications

Obelisk is a cloud-based Platform as a Service (PaaS) developed by IDLab [21]. It is jointly used by stakeholders from industry and academia to conduct collaborative research on IoT applications. These stakeholders include data producers, data scientists, application builders and end-users. The platform itself is presented as an intermediate layer between the different components and functions as the backbone within the overall Living Lab architecture, as visualized by Figure 1. It supports a number of different protocols and is used for stream processing within the platform tier or data consumption by enterprise tier applications, e.g., dashboard applications. However, the most important use of the platform is data persistence at large scale. Throughout this section, the internal architecture of Obelisk is discussed along with the important design choices made for each component.

The terminology used is derived from the IoT concept. A *Thing* is an all encompassing name for sensors and actuators. A single Thing can measure more than one type of measurement, named a *Metric*, and is addressable with an ID. It can be a single cyber-physical device that has multiple sensor-heads, e.g., vibration, temperature, as different metrics. A group of Things belonging to the same company, manufacturing plant, project, etc. are encapsulated within a *Scope*. Doing this provides a number of benefits with regard to maintainability, security and data isolation. This data isolation is ensured throughout the entire data flow, i.e., from ingest to egress. Permissions for both users and service accounts are limited to one or more *Scope(s)*, preventing unauthorized parties from gaining access to classified data. The fine-grained access control is based on standards such as OpenID [27].

### 5.1. Architecture

As Obelisk is presented as a cloud platform, the Application Programming Interface (API) is the main point of interaction with the system, which is composed of multiple services, each providing a key piece of the puzzle. As previously highlighted in Figure 1, The Obelisk API can be divided into three major parts: (i) The ingest API supports the data producers to securely and reliably insert data into the system; (ii) The egress API can be used to consume the events, both historical and in real-time, in order to process and/or visualize the data; (iii) Finally, the metadata API provides additional information or metadata about the available data for application builders and consumers. This includes information about user permissions and the available Scopes, Things and Metrics.

**Figure 3:**
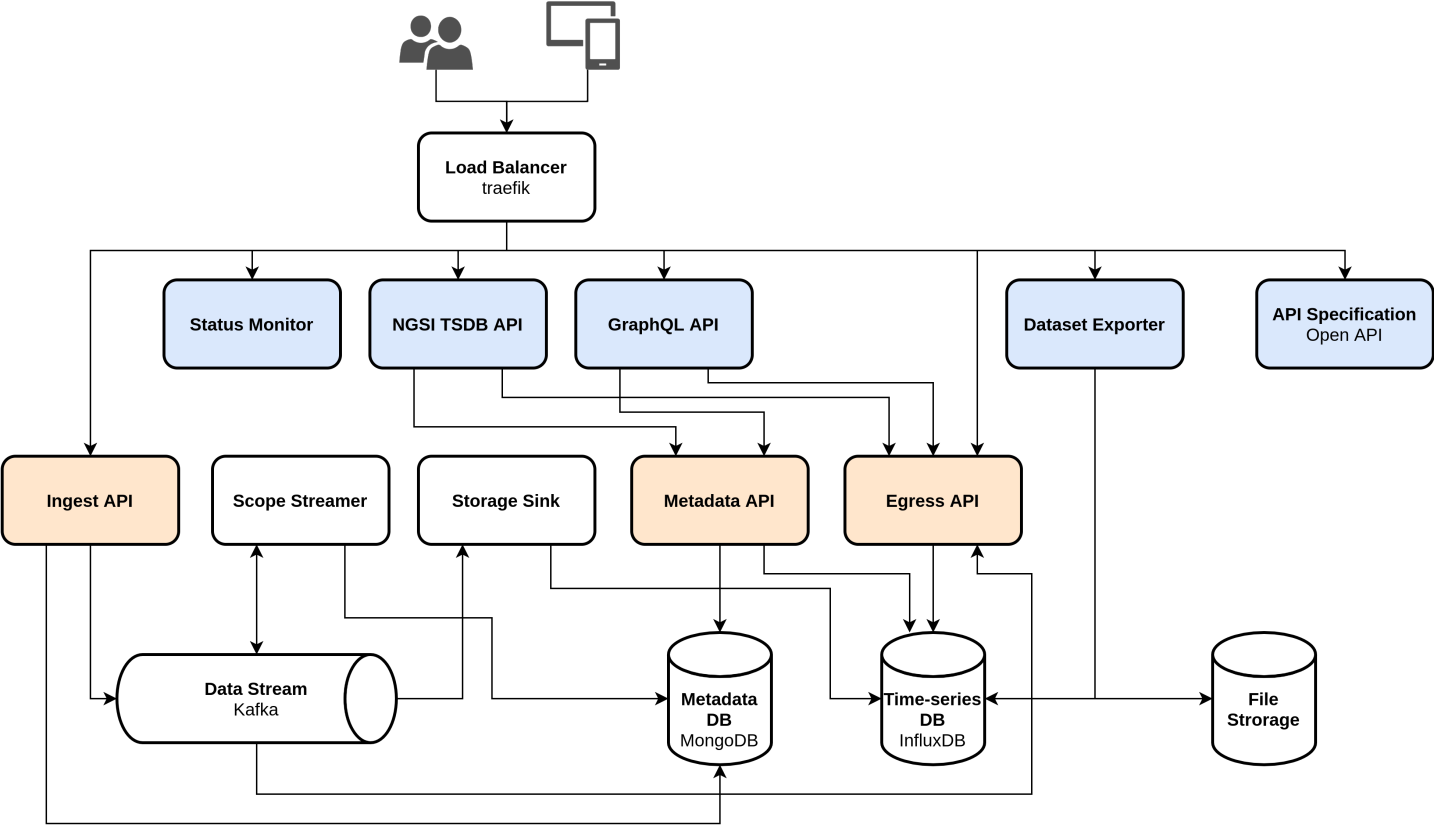
Obelisk architecture. Combined, the blue and orange components form the Public API for the stakeholders (users and connected devices), accessible through the load balancer. The arrows indicate dependencies between the different components.

When ingesting new data, it is pushed onto an internal data streaming platform. This component is highly critical since each incoming event passes through it. The decision for Apache Kafka was made due its high-throughput, low-latency, resiliency and scalability [28]. As shown in Figure 3, two internal components are listening to the message queue for incoming events: the *scope streamer* and the *storage sink*. The scope streamer routes these events to their respective scope related streaming pipeline, based on the available metadata, for consumption through the egress API as real-time events. The storage sink ensures data persistence by writing the incoming events to the time-series database.

As metadata database, the option was made for a NoSQL DB, namely MongoDB [29], as it requires no fixed underlying data schema. The biggest advantage of using a schema-less design in this scenario is its ability to scale horizontally. Additional database servers can be added, creating a MongoDB cluster consisting of one primary, or master, server and multiple secundary, or slave, servers. Using a cluster of database servers, read operations can then be executed on all members of the cluster. The write operations, however, are constrained to the primary server, but implementation of MongoDB’s sharding technique allows to parallelize this. Its ability to become larger and much more powerful through addition of database servers makes MongoDB the preferred choice for large and constantly evolving data sets. As time-series database, InfluxDB [30] was chosen. It uses the same core storage technology applied in most popular NoSQL databases today, including MongoDB, and thus possesses the same advantages with regard to flexibility, scalability and read/write speed.

As mentioned above, data consumption from Obelisk is achieved through the egress API. It consists of two sub-components each serving a different functionality. The first sub-component is the streaming API that supports real-time data consumption. This consumption of incoming data as Server-Sent Events (SSE) enables both stream processing by the RUL prediction services and real-time visualization of the data by enterprise tier applications such as the dynamic dashboard. The second sub-component is the RESTful API that can be queried to obtain historical observations and metadata. By using Obelisk as a middleware platform for stream processing, the machine learning services within our system are abstracted from the enterprise tier components such as the dynamic dashboard application. Improved or additional RUL prediction algorithms can therefore be deployed as new services within the overall system without inducing any changes to the dependent components.

To support different protocols, additional endpoints for Graph Query Language (GraphQL) and Next Generation Service Interface (NSGI) v2 [31] are added as adapters on top of the RESTful API. GraphQL is a lightweight query language developed by Facebook in 2016 and presented as an alternative for REST. One of its main advantages is the ability to define precisely the data you want, replacing multiple REST requests with a single call [32]. NGSI v2 is a data format used for context information management in IoT applications and is intended to manage the entire lifecycle of context information, including updates, queries, registrations, and subscriptions. A final option for data retrieval is by use of the dataset exporter. This component allows the file-based extraction of data in large quantities from the system.

### 5.2. Scalability

Obelisk has been designed with scalability in mind. This is apparent from the overall architecture, the technologies chosen, e.g., the streaming platform, the databases, and even the internal operation of individual components. Firstly, the architecture is in compliance with the Microservice-based Architecture (MSA) [33]. This indicates that each component presented in Figure 3 is a loosely-coupled, fine-grained service, or microservice that can be independently deployed. This architecture does not only improve the maintainability and continuous development, but also the scalability of the platform as a whole. Container-orchestration systems, e.g., Kubernetes [34], permit automatic scaling of individual services on-demand. The status monitor component shown in Figure 3 performs a complete data flow throughout the entire system to obtain an accurate health status of each service. By doing so, bottlenecks in the system can be detected early and quickly acted upon by replicating one or more components at the root of the problem.

Secondly, in addition to the scalable design, various strategies have been implemented at software level to protect against usage spikes. For instance, the ingest API applies a tuneable budget-based rate limiting, while the storage sink, shown in Figure 3, implements buffering strategies.

## 6. Dynamic Dashboard

The main point of contact for end-users with the presented IIoT system for fleet monitoring and visualization are enterprise tier applications. These enterprise tier dashboard applications combine data from different sources, queried from the platform tier, and present it to the user in an intuitive manner. For this living lab, the dynamic dashboard application developed by IDLab [26] enables end-users, e.g., operators, managers, to query the most relevant data from the information services, e.g., physical assets, and machine learning algorithms, by querying Obelisk and aggregating the data into meaningful visualizations accessible through a dynamic Graphical User Interface (GUI). The purpose of the dashboard is to ensure that the end-user can make business decisions in a timely manner based on the data. The in-house dashboard application distinguishes itself from commercial alternatives available today, e.g., Tableau, Grafana, through its automated sensor discovery and reduction of sensor and visualization choice overload by suggesting appropriate visualizations for selected sensors, using semantic reasoning on the available sensor and visualization metadata. Its aim is to eliminate as much of the configuration work as possible when setting up a new (fleet of) asset(s) in the edge tier and creating dashboards to visualize them.

### 6.1. Sensor Discovery

Within a Local Area Network (LAN), sensor discovery can be achieved using network discovery methods such as DNS-SD, mDNS, UPnP, or protocol suites, e.g., DLNA, zeroconf. Once an asset is added to the fleet and connected to the network through smart sensors, its IP address is automatically obtained from the DHCP server and broadcast to other devices within the LAN using one of the above aforementioned protocols. This methodology proves useful at the edge tier, where all devices are deployed within the same network. However, enterprise tier applications, such as the dashboard application, can be deployed outside of the edge tier networks. As described in more detail in [26], the most pragmatic solution for discovery of sensors within a remote network is by providing a root address for each sensor network at run-time. Then, upon HTTP GET request to that IP address and a predetermined TCP port, a discovery service responds with a list of the available services and how to reach them.

The Web Thing Model [35] proposes a standard for the API routes required to communicate this list of available services and provides a unified RESTful API to access the diverse, discovered sensors and services that come with heterogeneous communication protocols and data formats. The Web Thing Model describes a set of API endpoints that need to be implemented by a Web Thing, each with their own goal. A complete overview of these endpoints, or routes, and their descriptions is given in Table 2. In addition to these endpoints, the Web Thing Model also specifies a JSON model to convey information about the resources, e.g., actions, properties, things.

A Web Thing compliant with the Web Thing Model, also named an Extended Web Thing, can directly expose its sensors, or properties and actuators, or actions, through the root URL (*{wt}/properties* and *{wt}/actions* endpoints respectively). In contrast to this direct integration pattern, an option for the so called gateway integration pattern is also supported. This enables an Extended Web Thing to expose other Web Things through additional resources (*{wt}/things/{thingId}*), therefore serving as a gateway.

When the data from each Web Thing, such as assets within a fleet, is exposed by a RESTful API compliant with the Web Thing Model, client applications in the enterprise tier are capable of automatically discovering the available sensor properties and actions. These client applications are thus no longer burdened by the configuration of new assets and the annotation of the data types and formats of their sensor properties. Therefore, the end-user of these applications is no longer expected to have any technical knowledge about the fleet to visualize its sensor data. The drawback, however, is that the configuration must still be performed within the Web Thing API by a technical expert. As the Web Thing Model API is a lightweight specification and therefore easily implemented without extensive effort. Furthermore, the registration of new assets can be automated through local network discovery methods as discussed in this section.

### 6.2. Architecture

**Figure 4:**
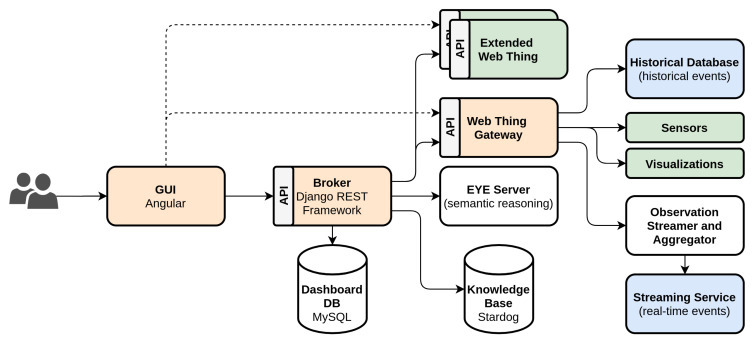
Dynamic Dashboard architecture. The orange components are core services within the dynamic dashboard, the blue components are provided by the platform tier, here Obelisk, and the green components are Web Things in the edge tier exposed according to the Web Thing Model.

As shown in Figure 4, the dynamic dashboard application consists of two core services: (i) the *Broker* component is the backbone for the dashboards as presented to the end-user. It provides functionality for authentication, data persistence, sensor discovery and semantic reasoning through its RESTful API. (ii) The *Web Thing Gateway* is used as an intermediate service that exposes an Web Thing Model compliant API for data sources that have not implemented the model themselves. These data sources are sensors, e.g., from the Things stored in Obelisk, and visualizations. The Web Thing gateway includes support for both historical events, through REST API calls, as well as real-time events by bridging the Obelisk SSE to the WebSockets protocol in conformity with the Web Thing Model. As shown in the architecture overview, the web thing gateway is only used in case the remote Web Things do not provide an API as specified by the Web Thing Model. Extended Web Things can be directly configured in the broker using its API or via the dashboard GUI.

Once the root URL of an Extended Web Thing or gateway is provided, the broker discovers the available properties and actions by crawling the available web resources on the API. Through a semantic extension of the Web Thing Model discussed in Section 6.1, semantic annotations of these resources, describing their purpose, can be retrieved using content negotiation through request headers, e.g., *Accept: application/ld+json* to retrieve semantic annotations in JSON-LD instead of the Web Thing Model’s standard JSON responses. This semantic context is then used by the broker to combine properties from multiple Web Things. To prevent the broker from intensively re-querying the Web Things, the semantic information is stored in a knowledge base. As pointed out earlier, not only sensors are presented as Web Things, also the visualizations used in the dashboard GUI are retrieved this way and they too are semantically annotated. By doing so, the dashboard broker is capable of semantic reasoning to match sensor properties to valid visualizations using the EYE server [26]. This partially automates the process of dashboard creation and assists the end-user in picking the most relevant visualization from the high number of supported visualization types.

### 6.3. Scalability

The dynamic dashboard application ensures scalability in various ways and aspects of the system. First and foremost, this application, similar to Obelisk, is designed according to the MSA standard. This implies that once again, each component is packaged and deployed as a containerized application. In case the dashboard is used as a PaaS, this ensures scalability when the amount of users and therefore the amount of created dashboards increases. Secondly, the dynamic dashboard application ensures scalability when the number of monitored assets within the system increases. Through the automated sensor discovery and (reasoned) dashboard visualization suggestions, the overhead for the end user remains minimal by removing the responsibility of manually adding and configuring new assets within the dashboard application as well as assisting these users in matching the new incoming data with the available visualizations. Lastly, the introduced impact on the network usage and Web Thing Gateways due to automated sensor discovery can be minimized by intelligent caching and scheduling strategies. By doing so, the sensor discovery can be planned during periods of minimal system load. This safeguards the network usage within the overall system when the amount of available Web Thing Gateways or created dashboards and therefore the requests made by the dashboard application to these components increases.

## 7. Results

The presented Smart Maintenance Living Lab architecture is currently deployed in practice and provides companies a scalable monitoring and visualization system for smart maintenance from edge to enterprise tier, from cyber-physical assets to dashboard, by solving the three research questions addressed in this paper:

1. Creation of a dataset with bearing faults under realistic and varying operating conditions;
2. Design of a robust, scalable and secure platform for sensor data ingestion and stream processing;
3. Design of a dynamic, intuitive dashboard application that reduces the amount of manual configuration required to construct the desired sensor data visualizations.

Regarding the first research question, free samples of the experimental dataset can be downloaded at https://www.flandersmake.be/en/datasets. These samples are part of a larger set for various operating conditions such as rotation speed, load and different fault types and severities.

**Figure 5:**
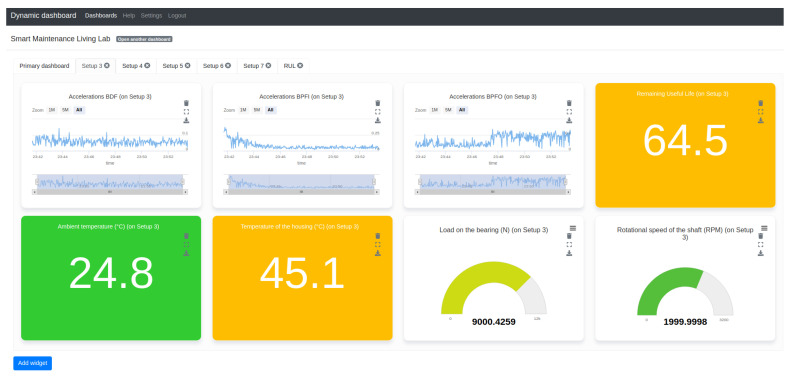
Visualization within the dynamic dashboard application of KPIs from Setup 3 in the Smart Maintenance Living Lab as listed in Table 1.

As for the latter two research questions, the complete platform is demonstrated and validated for industrial use cases. Therefore, an example dashboard has been created, as shown in Figure 5. This key component within the enterprise tier of the architecture is the main point of contact for the researchers working on, and the companies visiting, the living lab. It enables them to monitor the entire fleet of seven machines located at the edge tier by consuming their measurements and calculated features, as listed in Table 1, from the platform tier IoT middleware, Obelisk. The monitoring dashboard for the operator includes widgets for the seven most important KPIs. Due to the semantic reasoning within the dynamic dashboard application, the overhead and manual labour of creating and configuring these eight chosen widgets for each of the seven setups is reduced, resulting in a more user-friendly monitoring environment.

To demonstrate and validate the presented system for smart maintenance applications, a machine learning algorithm has been integrated with the platform tier. Through stream processing of the incoming data in Obelisk, a prediction of the RUL is calculated [36]. This percentage value indicates the estimated overall health of the bearing, 100% indicating that the bearing is completely new and 0% that the bearing has completely broken down. After real-time processing of the incoming data, these RUL predictions are pushed to Obelisk as data points of a RUL metric. Each of these data points is linked back to the setup or source from which it originates. By doing this, the dashboard can immediately consume these data as can be done with any other metric or KPI. Figure 5 contains a widget to monitor the RUL of the shown setup. At the captured moment in time, the bearing in this setup is flagged as degrading and is currently approximately at 64.5% of its remaining useful life. This provides feedback and can trigger an alert for the operator that maintenance will be required and should be scheduled in the near future.

## 8. Conclusions and Future Work

This paper proposed and implemented a complete architecture for IIoT applications. Throughout the design process of this architecture and its key components, special attention was given to the open challenges with regard to robustness, scalability and security that arose through adoption of the IoT concept in industry, resulting in a scalable system for fleet monitoring and visualization. This system has been demonstrated and validated though a smart maintenance use case, leading to an open test and research platform for accelerated bearing lifetime tests. However, this Smart Maintenance Living Lab is more than a proof-of-concept for smart maintenance applications in manufacturing industries. Through further data collection of end-to-life bearing tests, more intelligent and accurate machine learning algorithms can be created. This increase in accuracy will improve the overall performance of the presented system, further improving the PdM on machines consisting of rolling-element bearings and its benefits to industry.

Furthermore, each individual component within the system, i.e., the Obelisk cloud platform, the RUL estimation service and the dynamic dashboard application, is designed for reusability. Obelisk serves as a key component in various data science related research projects and can be applied to many IoT scenarios, such as smart cities, transportation and healthcare. It is continuously improved and new features are added on a regular basis. Next iterations of Obelisk will include more intelligent device management and support for various options for data ingestion with regard to data formats and communication protocols. In fact, the communication protocol can be completely abstracted through the use of adapters, supporting various state-of-the-art protocols common in IoT applications [37,38]. The dynamic dashboard used in the system is presented as complete dashboard solution. However, due to its modular design and adoption of the MSA, each key building block is encapsulated in a service. This enables the integration of the core functionality, e.g., sensor discovery, semantic visualization reasoning, in other dashboard applications, e.g., Grafana [39], Tableau [23], ThingsBoard [40]. Future work for the dynamic dashboard involves further automation and personalization of the dashboard creation process and therefore further reducing the manual overhead required of the end-user. This can be realised through use of a recommender system that is capable of selecting the best option from the list of valid visualizations retrieved from the semantic reasoner, taking into account the context and end-user preferences. This recommender system can then once again be packaged as a service and used by the aforementioned dashboard applications.

In conclusion, this paper aims to support the adoption of smart machine maintenance in industry through (i) the development of an open test and research platform to generate a valuable and otherwise difficult to obtain benchmark data set for the improvement of machine learning algorithms applied to smart machine maintenance and (ii) the design, implementation and validation of a complete architecture for IIoT applications with specific requirements concerning robustness, scalability and security and therefore reducing the reticence in the industry to widely adopt these technologies.

## Figures and Tables

**Table 1:** Overview of the measurements captured at the edge tier and features calculated by the edge data processor to reduce the volume of raw measurement data.

Feature	Description
**Raw measurements**	
load	Load on the bearing
rpm	Rotation speed of the shaft
temp.env	Ambient temperature
temp.housing	Temperature at bearing housing
**Statistical features**	
acc.RMS	Root Mean Square of acceleration signal
acc.kurtosis	Kurtosis of the acceleration signal
acc.peak	Peak of the acceleration signal
**Bearing fault features**	
acc.BDF	Bearing Defect Frequency
acc.BPFI	Ball Pass Frequency of Inner ring
acc.BPFO	Ball Pass Frequency of Outer ring

**Table 2:** The Web API endpoints that a Web Thing RESTful API must provide to make it compliant with the Web Thing Model.

Endpoint	Description
{wt}	The root resource URL.
{wt}/model	The model description of the Web Thing.
{wt}/properties	The list of available properties directly exposed by the Web Thing.
{wt}/properties/{propertyId}	The details of a specific property.
{wt}/actions	The list of available actions, in case the Web Thing can control actuators, e.g., can adjust rotation speed.
{wt}/actions/{actionId}	The details of a specific action.
{wt}/actions/{actionId}/{executionId}	Execution of a specific action.
{wt}/things	Lists additional Web Things that the Web Thing exposes when functioning as a gateway.
{wt}/things/{thingId}	A specific Web Thing exposed by the current Web Thing.
{wt}/subscriptions	The list of subscriptions to actions or properties, by clients that want to be notified when the state of this action or property changes.

## Abbreviations

The following abbreviations are used in this manuscript:

API	Application Programming Interface
CM	Corrective Maintenance
CPS	Cyber-Physical System
DHCP	Dynamic Host Configuration Protocol
DLNA	Digital Living Network Allicance
DNS	Domain Name System
DNS-SD	DNS Service Discovery
EYE	Euler Yet another proof Engine
GUI	Graphical User Interface
HTTP	HyperText Transfer Protocol
IEEE	Institute of Electrical and Electronics Engineers
IIoT	Industrial Internet of Things
IP	Internet Protocol
IoT	Internet of Things
JSON	JavaScript Object Notation
JSON-LD	JSON for Linked Data
KPI	Key Performance Indicator
LAN	Local Area Network
LoRaWAN	Long Range Wide Area Network
mDNS	multicast DNS
MSA	Microservice-based Architecture
NGSI	Next Generation Service Interface
PaaS	Platform as a Service
PdM	Predictive Maintenance
PHM	Prognostics and Health Managmeent
PM	Preventive Maintenance
REST	REpresentational State Transfer
RUL	Remaining Useful Life
SMM	Smart Machine Maintenance
SQL	Structured Query Language
SSE	Server-Sent Events
TCP	Transmission Control Protocol
UPnP	Universal Plug and Play
URL	Uniform Resource Locator
VNO	Virtual Network Operator
